# Supplementary material for: Integrating genomic structural equation modeling and experimental validation to unravel the genetic basis of male genital lichen sclerosus
Source: Front Immunol. 2026 Jul 16;17:1857945. doi: 10.3389/fimmu.2026.1857945 (PMC13435250; doi:10.3389/fimmu.2026.1857945)
Supplement: Supplementary file 6 [file SupplementaryFile5.zip › result/fuma_PSYCH_factor_sCCA1_focus.chr17.chr17.77298636.78837588.pop1.pdf]

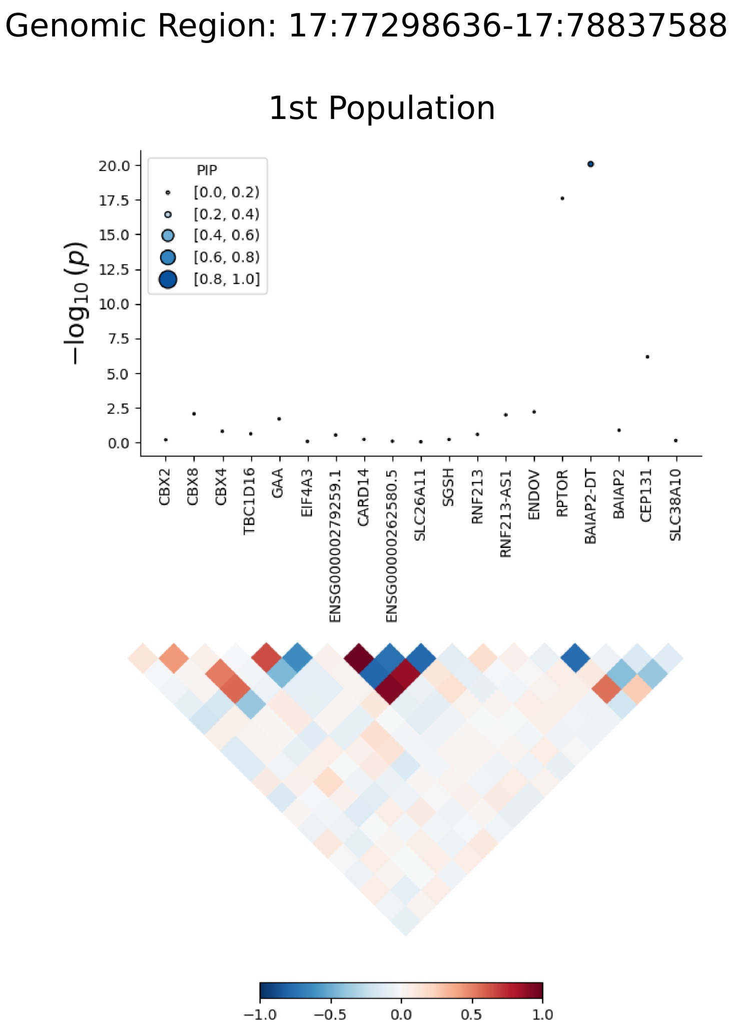

Genomic Region: 17:77298636-17:78837588
1st Population
PIP
[0.0, 0.2)
[0.2, 0.4)
[0.4, 0.6)
[0.6, 0.8)
[0.8, 1.0]
$-\log_{10}(p)$
20.0
17.5
15.0
12.5
10.0
7.5
5.0
2.5
0.0
CBX2
CBX8
CBX4
TBC1D16
GAA
EIF4A3
ENSG00000279259.1
CARD14
ENSG00000262580.5
SLC26A11
SGSH
RNF213
RNF213-AS1
ENDOV
RPTOR
BAIAP2-DT
BAIAP2
CEP131
SLC38A10
−1.0
−0.5
0.0
0.5
1.0
